# Main Oral Manifestations in Immune-Mediated and Inflammatory Rheumatic Diseases

**DOI:** 10.3390/jcm8010021

**Published:** 2018-12-25

**Authors:** Roberta Gualtierotti, Angelo Valerio Marzano, Francesco Spadari, Massimo Cugno

**Affiliations:** 1 Department of Clinical Sciences and Community Health, Università degli Studi di Milano, 20122 Milano, Italy; 2 Department of Pathophysiology and Transplantation, Università degli Studi di Milano, Dermatology Unit, IRCCS Fondazione Ca’ Granda, Ospedale Maggiore Policlinico, 20122 Milano, Italy; angelo.marzano@unimi.it; 3 Department of Biomedical, Surgical and Dental Sciences, Università degli Studi di Milano, 20122 Milano, Italy; francesco.spadari@unimi.it; 4 Department of Pathophysiology and Transplantation, Università degli Studi di Milano, Internal Medicine, IRCCS Fondazione Ca’ Granda, Ospedale Maggiore Policlinico, 20122 Milano, Italy; massimo.cugno@unimi.it

**Keywords:** rheumatoid arthritis, systemic lupus erythematosus, Behçet’s disease, Sjögren’s syndrome, oral lesions

## Abstract

Oral manifestations are frequent in patients with rheumatic diseases. The aim of this review is to offer readers practical advice concerning the onset, diagnosis and treatment of the main oral manifestations encountered in rheumatological and dental clinics. Signs and symptoms such as oral hyposalivation, xerostomia, temporomandibular joint disorders, periodontal disease, and dysphagia may be the first expression of a number of rheumatic diseases. Some of these manifestations are aspecific and very frequent, such as oral aphthosis, which can be the first manifestation in patients with systemic lupus erythematosus; some are potentially dangerous, such as jaw claudication during the course of giant cell arteritis; and some are very rare but peculiar, such as strawberry-like gingivitis in patients with granulomatosis with polyangiitis. Other oral manifestations are due to adverse reactions to disease-modifying anti-rheumatic drugs. Oral alterations in rheumatic diseases are frequently overlooked in clinical practice, but their prompt recognition not only allows the local lesions to be appropriately treated, but also makes it possible to identify an underlying systemic disease.

## 1. Introduction

It is estimated that about 3% of the population suffer from a chronic inflammatory rheumatic disease (IRD) [1], and many of these patients experience oral manifestations, which may be the first clinical sign or symptom of a systemic disease (Table 1). Manifestations such as oral aphthosis are non-specific and very frequent, but their presence in association with typical clinical manifestations and disease biomarkers should give rise to clinical suspicion: oral aphthous ulcers have a prevalence of up to 50% of patients with systemic lupus erythematosus (SLE) [2,3], whereas xerostomia and hyposalivation are respectively a symptom and sign reported by 90% of patients with Sjögren’s syndrome. A few oral manifestations are very rare but typical, such as strawberry-like gum disease in patients with granulomatosis with polyangiitis. Patients with systemic sclerosis (SSc) often have microstomia and microcheilia that may affect mouth opening and thus interfere with their everyday activities and quality of life. Finally, some oral presentations are potentially dangerous such as jaw claudication as an expression of giant cell arteritis. The optimal management of such oral manifestations requires a multidisciplinary team of dentists and rheumatologists. 

Here, we review the main oral manifestations of rheumatic diseases, and provide the reader with a practical advice concerning the onset, diagnosis and treatment of these lesions. We searched the PubMed MEDLINE database for English language articles published since 1980 that referred to stomatological/oral/orofacial lesions in inflammatory rheumatic disease. We included articles regarding patients with rheumatoid arthritis, juvenile idiopathic arthritis, systemic lupus erythematosus, Sjögren’s syndrome, systemic sclerosis, immune-mediated myopathies, Behçet’s disease, giant cell arteritis, or granulomatosis with polyangiitis. The selected articles were identified by specialists in rheumatology, internal medicine, dermatology and dentistry, based on their expertise. Any articles for which no full text was available were excluded.

## 2. Oral Manifestations in Rheumatic Diseases

### 2.1. Microstomia/Microcheilia

Patients with microstomia (i.e., reduced oral aperture) may experience limitations in everyday activities such as eating and oral hygiene. SSc should be suspected when microstomia and microcheilia (i.e., small lips) are associated with face, mucosal and body telangiectasias (i.e., macroscopically visible dilated skin vessels), typical perioral radial folds, Raynaud’s phenomenon (i.e., a recurrent vasospasm of the fingers and toes, generally followed by a cyanotic and erythematous phase usually occurring in response to stress or cold temperature), and skin thickening.

SSc is a chronic systemic disease of unknown etiology that is characterized by vasculopathy and fibrosis of the skin and internal organs which, if left untreated, may lead to irreversible organ failure. It is a rare disease, with an estimated prevalence of approximately 250 per million and a female:male ratio of 7:1 [4,5]. Typical autoantibodies directed against centromeric proteins (CENPA/B), Scl70-topoisomerase I, RNA polymerase III, and U1 ribonucleoprotein can be detected in the great majority of patients. The spectrum of clinical manifestations ranges from Raynaud’s phenomenon (present in almost all patients) to life-threatening complications such as pulmonary arterial hypertension and interstitial lung fibrosis [6]. The progression of vascular and fibrotic organ damage leads to chronic morbidity and a high mortality rate [7]. The differential diagnosis of SSc includes immunological or inflammatory diseases (e.g., chronic graft versus-host disease, eosinophilic fasciitis), metabolic diseases (e.g., acromegaly, amyloidosis), inherited diseases (e.g., phenylketonuria, porphyrias) and localized diseases (e.g., idiopathic pulmonary fibrosis, sarcoidosis, esophageal hypomotility syndromes) [8].

Although oral alterations in patients with SSc are responsible for major functional disabilities affecting everyday life, they are often overlooked. A web-based survey by Leader et al. found that about 50% of dentists felt they might cause harm to SSc patients because of their insufficient knowledge of the disease [9]. Microstomia, which limits mouth opening in 70% of SSc patients, is the most frequent oral finding and is due to fibrosis of perioral soft tissue [10]. Subcutaneous collagen deposition in facial skin gives the face a characteristic smooth, mask-like appearance, and may be associated with perioral, labial or tongue telangiectasias.

The treatment of SSc depends on the localization and severity of the disease, and includes general immunosuppression and the use of disease-modifying anti-rheumatic drugs (DMARDs) such as mycophenolate mofetil, methotrexate and cyclophosphamide to treat specific complications. Biological agents such as tocilizumab and rituximab have also been used, but the results are controversial [7]. A multidisciplinary approach should be adopted to encourage oral hygiene and rehabilitation [29]. Given the high incidence of tongue carcinoma among SSc patients [30], the oral mucosa should be systematically examined on a regular basis so that early lesions can be promptly identified and treated [29]. Raynaud’s phenomenon in the tongue has also been described [31]. Patients with clinical manifestations suggesting SSc who are first seen by a dentist need to be referred to a rheumatologist as soon as possible because there is evidence that the progression of organ involvement is rapid in the earlier stages of the disease [7]. Furthermore, patients presenting with clinically advanced pulmonary arterial hypertension have a much worse prognosis than those identified earlier in the course of SSc [32,33]. The management of microstomia and microcheilia is less clear: Exercises for improving mouth opening have been proposed and fat grafting has led to promising results, but further evidence is needed [10,34,35].

### 2.2. Dysphagia

Motility during swallowing is achieved by combining smooth muscle activity within the esophageal wall with the action of striated muscles in the oropharynx: Dysphagia (a subjective difficulty in swallowing liquids or solids) may be due to an impairment in either mechanism or both [23]. Oropharyngeal dysphagia may arise from disorders affecting the function of the oropharynx and upper esophageal sphincter, whereas esophageal dysphagia may arise from disorders involving the body of the esophagus or the lower esophageal sphincter.

Esophageal involvement is present in up to 90% of SSc patients [21] and, in addition to dysphagia, may also give rise to retrosternal burning, gastroesophageal reflux, esophageal strictures, and erosive esophagitis, all of which are usually due to a combination of neural dysfunction and the fibrotic replacement of muscle. Esophageal manometry has revealed decreased activity in the lower third of the esophagus and a hypotonic lower esophageal sphincter in SSc patients [36]. The treatment of esophageal dysmotility and gastroesophageal reflux is respectively based on the use of prokinetic agents and proton pump inhibitors.

In patients with immune-mediated myopathies, impairments in the skeletal muscles of the posterior pharyngeal wall and proximal third of the esophagus can lead to oropharyngeal dysphagia and, in some cases, dysphonia (i.e., an alteration in the quality of the voice) [23]. Immune-mediated myopathies are a heterogeneous group of systemic inflammatory myopathies that include dermatomyositis, polymyositis, inclusion-body myositis and immune-mediated necrotising myopathy [37]. Their incidence is not very well documented, but it is known that polymyositis and dermatomyositis are very rare diseases whose estimated overall incidence is 1 to 10 new cases per million people per year [23]. They are characterized by muscle weakness, high creatine kinase levels, myopathic electromyographic findings, and the involvement of organs such as the skin, joints, lungs, and the gastrointestinal and cardiovascular systems. Anti-nuclear antibodies (ANAs) are very frequently observed and are used for screening purposes. Myositis-associated autoantibodies include anti-3-hydroxy-3-methylglutaryl coenzyme A reductase (HMGCR), anti-cytosolic 50-nucleotidase 1A, anti-Mi-2 nuclear antigen, anti-transcriptional intermediary factor 1-g (TIF-1g), anti-melanoma differentiation-associated gene 5, anti-small ubiquitin-like modifier activating enzyme, anti-nuclear matrix protein 2, anti-Jo1-histidyletransfer RNA synthetase, and anti-signal recognition particle [38]. Dermatomyositis and necrotizing autoimmune myopathy are associated with an increased risk of cancer in comparison with the general population, particularly in the presence of antibodies such as anti-HMGCR and anti-TIF-1g [38]. A muscle biopsy often reveals inflammatory infiltrates [39].

Dysphagia has been reported in 12% to 54% of patients with polymyositis and dermatomyositis [40]. Oropharyngeal striated and esophageal smooth muscles can both be affected. Studies have shown an increase in pharyngeal transit time from the oral cavity to the upper esophageal sphincter [41]. Dysphagia in patients with dermatomyositis has been associated with an increased incidence of internal malignancies [42]. Patients with impaired deglutition may also complain of hypersalivation due to a combination of impaired swallowing muscles and the salivary reflex caused by reflux. Muscular symptoms may be associated with constitutional symptoms such as fatigue, low-grade fever, weight loss, and arthralgia or arthritis [43,44].

Treatment of the systemic disease with glucocorticoids and DMARDs such as methotrexate, azathioprine, intravenous immunoglobulins, cyclosporine and mycophenolate mofetil is usually effective in controlling disease progression [38]. Rituximab has also been successfully used in both adult and pediatric patients with refractory myositis [45]. Myotomy may be effective in the case of cricopharyngeal sphincter involvement when conservative treatment has failed [46].

### 2.3. Aphthosis 

Recurrent oral aphthous ulcers are a frequent clinical manifestation in patients with IRDs, and are particularly frequent in patients with SLE; they are therefore included in both the 1987 American College of Rheumatology and the 2012 Systemic Lupus International Collaborating Clinics SLE classification criteria [47,48]. SLE is the prototype chronic systemic autoimmune disorder: It is characterized by autoantibody production, immune complex deposition and complement activation, which may affect any bodily organ, and is associated with a plethora of clinical and immunological abnormalities [49]. SLE has an incidence of 3.8 per 100,000 per year and a prevalence of 26.2 per 100,000 in the UK, a female:male ratio of about 9:1, and predominantly affects women of childbearing age [50]. ANAs are present in up to 90% of patients, and double-stranded DNA (dsDNA) autoantibodies in up to 70%.

The reported prevalence of oral aphthous ulcers among SLE patients is about 50% over a lifetime and examinations usually reveal more than one lesion [2,3,17]. The differences in the reported rates may be due to the fact that oral examinations do not always form part of a routine rheumatological examination. The aphthous ulcers classically appear as a whitish plaque with central erythema and peripheral keratotic striae [17], but other oral lesions such as lichenoid mucositis and discoid lesions, which may localize in buccal and gingival mucosae and the lips have also been reported [17,51]. Direct immunofluorescence often reveals linear deposits of immunoglobulin (Ig) G or IgM and/or C3 in the basement membrane. CD4+ T lymphocytes are the prevalent cell subtype, but other immune cells may also be present [17,52]. 

Treatment is based on corticosteroids and DMARDs such as azathioprine, cyclosporine, methotrexate or mycophenolate mofetil, belimumab and off-label rituximab in refractory cases. Systemic therapy may reduce the prevalence of oral aphthous ulcers in treated patients. The local management of aphthous ulcers includes the use of non-steroidal anti-inflammatory mouth washes and steroidal rinses, gels and ointments [51].

Behçet’s disease is a systemic vasculitis characterized by oral aphthous ulcers, genital ulcers, ocular lesions and systemic manifestations such as erythema nodosum and pseudofolliculitis, life-threatening cardiovascular and neurological involvement, and venous and arterial thrombosis [24,25]. Its complex etiology is not fully understood, but the currently accepted hypothesis is an autoimmune process triggered by an infectious or environmental agent in genetically predisposed subjects, although an autoinflammatory pathogenesis has also been suggested [25,53]. It is a rare disease that is more prevalent along the ancient trading route known as the “Silk Road” between eastern Asia and the Mediterranean area. The human leukocyte antigen (HLA)-B51 allele located on chromosome 6p is the most closely associated risk factor for the disease [25].

**Figure 1:**
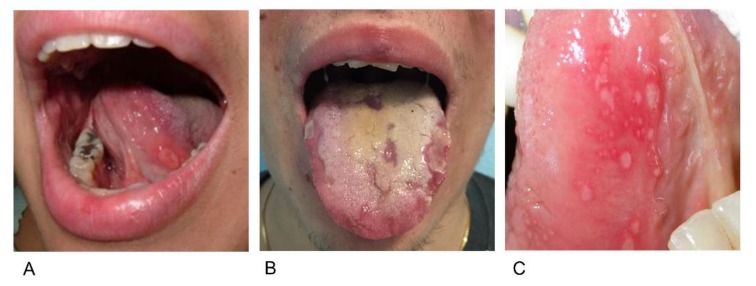
Oral ulcers in patients with Behçet’s disease: (**A**) A centimetric aphthous lesion on the left ventral side of the tongue; (**B**) major deep aphthae on both sides of the tongue; and (**C**) aphthous lesions with a herpetiform distribution on the ventral side of the tongue.

There are three clinical variants of the recurrent oral aphthous ulcers associated with Behçet’s disease: Minor aphthae (<10 mm in diameter), major aphthae (>10 mm in diameter, deeper than minor ulcers and painful) and herpetiform ulcers (numerous clustering pinpoint ulcers) (Figure 1A–C). The aphthae are managed by means of topical or systemic treatment with corticosteroids or systemic treatment with colchicine, or immunomodulatory or immunosuppressive drugs such as azathioprine, thalidomide, interferon-alpha and tumor necrosis factor alpha inhibitors [54,55].

**Figure 2:**
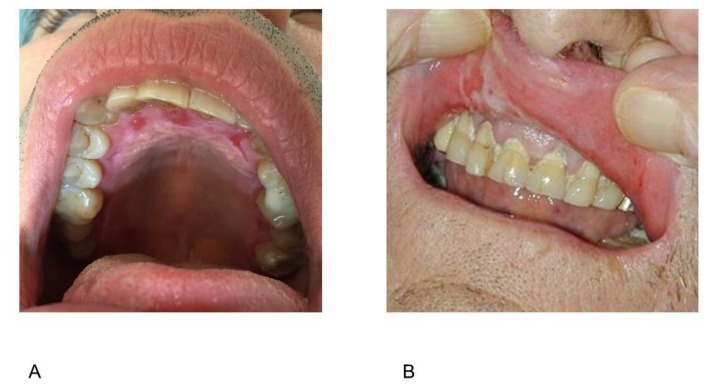
Oral involvement in patients with bullous diseases may manifest itself as erosions of the hard palate on a desquamative and hyperplastic background as in mucous membrane pemphigoid (**A**) or a wide erosion covered by a whitish pseudomembrane on the internal mucosa of the upper lip due to a ruptured bulla in bullous pemphigoid (**B**).

The differential diagnosis of oral ulcers includes disorders other than IRD, such as systemic autoimmune diseases, autoinflammatory diseases, infections, and adverse drug reactions. Inflammatory bowel diseases—particularly Crohn’s disease—and celiac disease may manifest themselves in the form of oral aphthosis [56,57]. Aphthous-like oral ulcerations may also appear in the case of autoinflammatory diseases such as familial Mediterranean fever (FMF); periodic fever, aphthous stomatitis, pharyngitis and adenitis (PFAPA); hyperimmunoglobulinemia D with periodic fever syndrome; tumor necrosis factor receptor-associated periodic syndrome; and pyogenic sterile arthritis, pyoderma gangrenosum, acne (PAPA) [58]. Aphthous ulcers in IRD patients are not preceded by blisters, but these may be present in patients with immunobullous diseases such as mucous membrane pemphigoid, which is characterized by bullae that rapidly evolve into irregular erosions that are typically associated with desquamative gingivitis extending to the hard palate (Figure 2A). Blistering and erosive lesions also indicate mucosal involvement in about 20% of patients with bullous pemphigoid (Figure 2B) [59]. Intra-oral herpes simplex virus (HSV) infection often involves keratinized surfaces such as the hard palate and attached gingival mucosa, which are spared in patients with aphthous ulcers; HSV infection can be identified by means of a polymerase chain reaction swab.

Methotrexate (MTX) is a folic acid antagonist that is used in chemotherapy and as an immunosuppressant in IRD treatment. When used to treat malignancies, high doses of MTX interfere with the synthesis of DNA bases by inhibiting folate pathway enzymes, particularly dihydrofolate reductase [60]. When used at lower doses as a DMARD, its anti-inflammatory effect is probably due to the fact that it increases the extracellular levels of adenosine, an anti-inflammatory mediator [61]. One study has found that the prevalence of stomatitis was higher in RA patients taking MTX than in those not taking it (37% vs. 19%) [62], but there were no patients receiving folic or folinic acid in either group. Other frequent causes of aphthous ulcers in IRD patients taking MTX are errors or misunderstandings in the administration schedule or dose, which usually ranges from 7.5 to 20 mg weekly [63]. Clear instructions and regular follow-up appointments may reduce the incidence of this adverse event [64]. Folic acid (also known as vitamin B9) is a synthetic form of folate, whereas folinic acid (5-formyl tetrahydrofolate) is found naturally in foods and is converted into any of the other active forms of folate in the body. Folinic acid supplementation is effective and does not interfere with the mechanism of action of MTX when taken 24 h after MTX [65]. There is no evidence of any significant difference between folic or folinic acid prophylaxis, but the low cost of folic acid makes it more cost-effective [66].

### 2.4. Xerostomia

Xerostomia refers to the symptom of dry mouth reported by patients, whereas hyposcialia is an objective reduction in salivary flow demonstrated by means of scialometry or other instrumental investigations such as salivary gland scintigraphy with (99 m)Tc-pertechnetate. Sjögren’s syndrome is a chronic autoimmune disease that typically targets exocrine glands. Its major clinical characteristics are oral and ocular dryness, but it is also often associated with systemic symptoms and signs. It is classified as primary in the absence of any other systemic autoimmune diseases, and secondary in the presence of another connective tissue disease (CTD), particularly RA or SLE [20,67].

Primary Sjögren’s syndrome mainly affects women in the fifth or sixth decade of life (its prevalence among women is 0.1% to 0.4% in the UK), although up to 10% of cases occur in men and it has also been reported in children [20,68]. Patients usually first consult primary care physicians, ophthalmologists or dental practitioners, but many of these lack specific knowledge.

**Figure 3:**
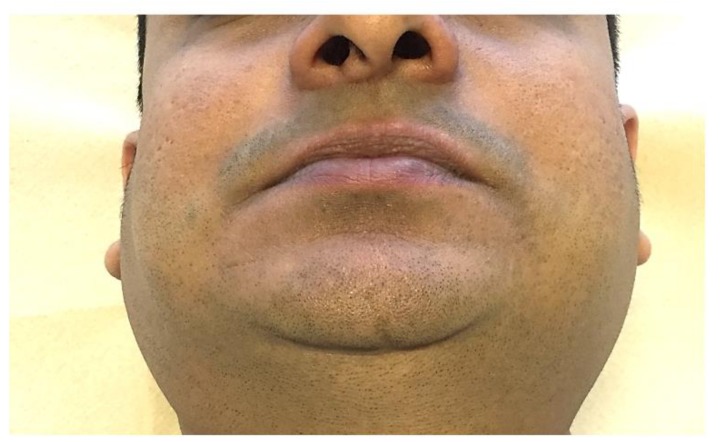
Parotid gland swelling during the course of Sjögren’s syndrome.

Sjögren’s syndrome may present as oral dryness and episodes of salivary gland swelling due to inflammatory infiltrate (Figure 3). In comparison with the general population and other CTDs, Sjögren’s syndrome is associated with a higher risk of developing non-Hodgkin lymphoma (NHL), particularly low-grade marginal zone lymphoma of mucosa-associated lymphoid tissue (MALT), diffuse B cell lymphoma (DBCL) or lymphoplasmacytoid lymphoma [69,70].

**Figure 4:**
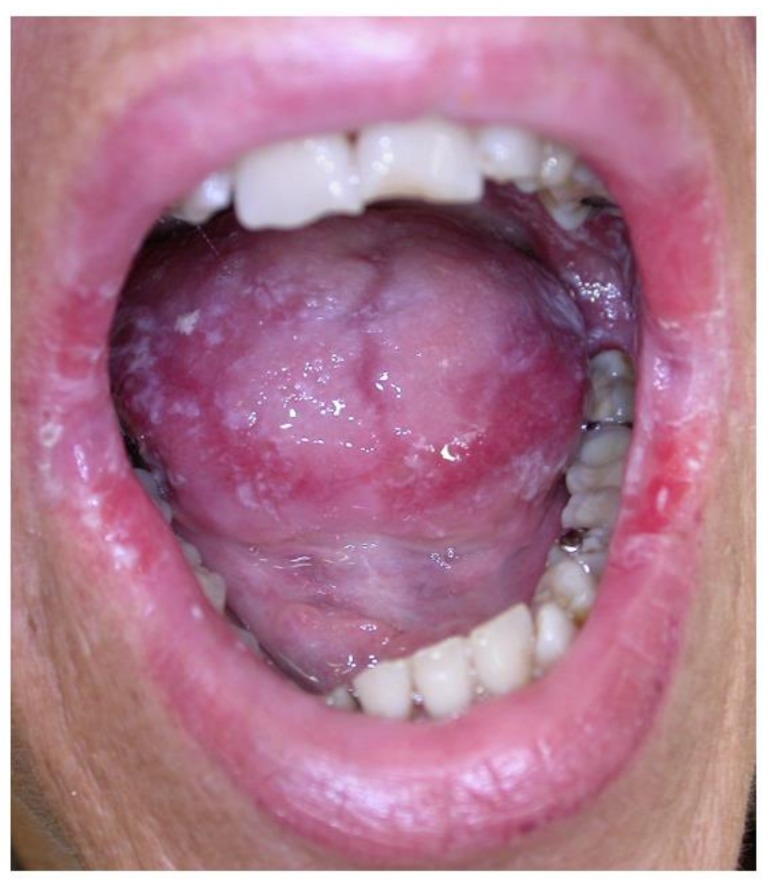
Chronic candidiasis of the oral mucosa with pseudomembranous, erythematous lesions and angular cheilitis during the course of Sjögren’s syndrome.

Many subjects report some degree of usually transient or mild oral dryness, but objective hyposcialia may give rise to dysarthria, dysphagia, dysgeusia, mucosal injury, an increased risk of tooth decay, gingivitis (but there is no clear evidence of a higher risk of periodontitis), and candidiasis (pseudomembranous, erythematous candidiasis and angular cheilitis), thus greatly affecting the patients’ quality of life (Figure 4).

The most widely accepted classification criteria for primary Sjögren’s syndrome are those of the American-European Consensus Group (AECG), which require the presence of four of the following criteria: (1) Dry eye symptoms; (2) dry mouth symptoms; (3) objective ocular dryness (Schirmer’s test <5 mm in 5 min or van Bijsterveld score); (4) objective oral dryness (unstimulated salivary flow rate <0.1 mL/min or positive salivary scintigraphy and sialography); (5) positive anti-SSA/Ro or anti-SSB/La antibodies; and (6) focal lymphocytic sialadenitis in a labial gland biopsy, with at least one of the last two objective features [71]. The American College of Rheumatology (ACR) and European League Against Rheumatism (EULAR) have recently developed new criteria based on the weighted sum of five items (autoantibodies, histological features, abnormal ocular staining scores, Schirmer’s test and unstimulated salivary flow rate) [72].

The typical autoantibodies in primary Sjögren’s syndrome are anti-SSA/Ro and anti-SSB/La antibodies, which are routinely identified as part of extractable nuclear antigen (ENA) laboratory screening. About two-thirds of patients with primary Sjögren’s syndrome have anti-SSA/Ro and/or anti-SSB/La antibodies [20]. Differential diagnosis should include other conditions that have similar or overlapping clinical features such as a history of head and neck radiation treatment, active hepatitis C virus infection, acquired immune deficiency syndrome, amyloidosis, graft-versus-host disease, and sarcoidosis [72,73]. 

IgG4-related disease (a rare systemic fibroinflammatory disorder that was first described in the 2000s and also includes disorders formerly regarded as different entities such as Mikulicz disease, Küttner’s tumor, Riedel thyroiditis, and Ormond disease) is also frequently associated with salivary gland swelling and xerostomia. It is characterized by high serum IgG4 levels and multi-organ inflammation that may target the salivary and lacrimal glands, periorbital structures, the pituitary gland, thyroid, pancreas, biliary tract, lungs, prostate gland and retroperitoneal cavity [74]. The classification criteria proposed by Umehara et al. in 2012 are based on the bioptic presence of IgG4+ cells and high serum IgG4 levels [75].

Drug-related xerostomia may be caused by many medications: Anti-cholinergic and/or sympathomimetic drugs, tricyclic antidepressants, benzodiazepines, diuretics, anti-histamines, opioids, selective serotonin reuptake inhibitors, proton-pump inhibitors and anti-HIV protease inhibitors.

Additional causes of xerostomia are dehydration, allergy and/or atopy and local infections, sarcoidosis, tuberculosis and amyloidosis [20].

The management of xerostomia involves the use of symptomatic treatment with saliva replacement and salivary gland stimulants (sialagogues). Sugar-free chewing gum is widely used and can be very helpful in dentate patients. Pilocarpine is a systemic muscarinic agonist that is effective in the presence of functional acinar tissue, but its side effects include sweating, abdominal discomfort and arrythmias, and may compromise patient compliance. Cevimeline is a selective muscarinic agent that acts on M3 receptors and consequently has a lower incidence of side effects, but it is not available in Europe. DMARDs are not recommended on a routine basis, but hydroxychloroquine may be effective in controlling oral symptoms, particularly in the presence of systemic features. The possibility of using biological agents to treat such features (including lymphoma) is currently being investigated, and there is growing interest in anti-B cell agents such as rituximab, belimumab and abatacept, which interfere with T and B cell interactions [76].

### 2.5. Gingival Overgrowth

Gingival overgrowth is the enlargement of attached gingiva due to an increased number of cells and the excessive accumulation of components of the extracellular matrix (ECM). The underlying molecular mechanisms are still unclear but, although it may simply be a consequence of gingivitis due to poor oral hygiene, it can also be associated with many systemic conditions [77].

One typical form of gingival overgrowth is strawberry-like gingivitis, which is characterized by reddened, swollen and somewhat granular tissue that is often covered with petechiae and small punctate lesions. It is an almost pathognomonic oral manifestation of granulomatosis with polyangiitis (once known as Wegener’s granulomatosis) [27,28], whose typical presentation has recently been described by Ghiasi [27]. It is a very rare necrotizing granulomatous vasculitis (estimated incidence 9.8 per million per year) [78] that typically affects small and medium-sized vessels. The most frequently involved site is upper respiratory tract, which is involved in 90% of cases at diagnosis and is characterized by granulomatous sinusitis resistant to the standard sinusitis treatment regimens. Early referral to an otorhinolaryngologist and rheumatologist is essential because, if left untreated, it may lead to the progressive destruction of maxillary and mandibular bone and nasal cartilage, nasal septum perforation, saddle nose deformity, damage to the walls of the sinus and orbit, and fistula formation. Furthermore, granulomatosis with polyangiitis may have life-threatening complications such as lung and renal involvement, with the former being typically characterized by alveolar hemorrhage, and the latter by necrotizing glomerulonephritis [79]. Other oral manifestations include mucosal ulceration with non-specific histology findings, and lingual necrosis, which has also been reported as a rare presenting sign [80]. Anti-neutrophil cytoplasm antibodies (ANCAs) are present in 90% of patients, particularly with the cytoplasmic fluorescence pattern of anti-PR3-ANCA [79]. The mainstay of the treatment of granulomatosis with polyangiitis is a combination of glucocorticoids and cyclophosphamide to induce remission, followed by maintenance treatment with azathioprine and methotrexate [79]. Rituximab has recently been approved for the induction and maintenance treatment of ANCA-associated vasculitides [81,82].

**Figure 5:**
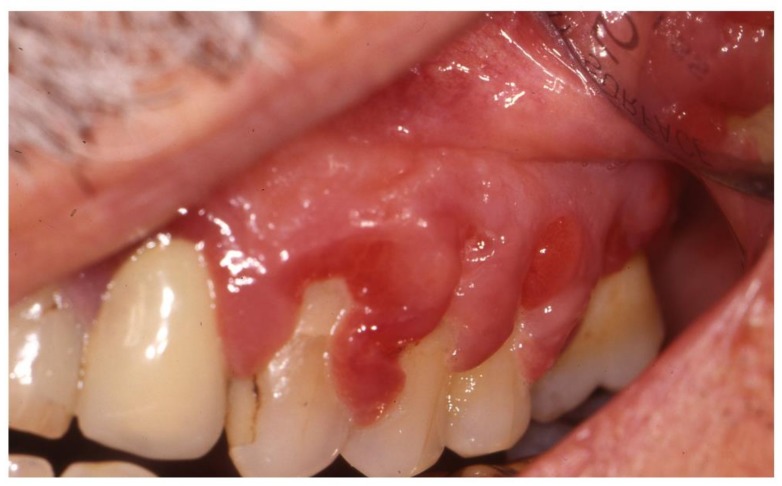
Gingival overgrowth caused by cyclosporine in a patient with psoriatic arthritis.

The differential diagnosis of gingival overgrowth includes hematological malignancies, granulomatous diseases, genetic disorders and drug-induced gingival overgrowth [77]. Gingival infiltration may be the first clinical manifestation of leukemia, and has been described in more than 60% of cases of acute monocytic leukemia and about 20% of cases of acute myelomonocytic leukemia [83]. Possible causes of granulomatous lesions include sarcoidosis, Crohn’s disease and tuberculosis, whereas the most frequent genetic disorder causing gingival overgrowth is hereditary gingival fibromatosis [77]. Drug-induced overgrowth should be considered in IRD patients taking antiepileptic drugs, calcium-channel blockers, and cyclosporine A (Figure 5) [77], and patients should be informed of this possible side effect, which usually resolves after a dose reduction or drug discontinuation. Switching to alternative drugs can also be considered: For example, tacrolimus is associated with a lower incidence and later onset of less severe gingival overgrowth than cyclosporine A [84].

As gingival overgrowth is worsened by plaque deposition on the teeth, patients should be encouraged to practice regular and thorough oral hygiene. In refractory cases, treatment with antibiotics such as azithromycin is effective in reducing cyclosporine-induced gingival hyperplasia [85]. Alternatively, the surgical or laser removal of excess gingiva (gingivectomy) may be performed, although the condition is likely to recur [67,86].

### 2.6. Periodontal Disease 

Periodontal disease is a chronic inflammatory condition caused and perpetuated by dysbiosis of the commensal microbiota in dental plaque that can destroy the gingiva and tooth-supporting tissues (bone and periodontal ligaments) and eventually leads to tooth loss [87,88]. The interactions of infectious, environmental and genetical factors and the host immune system give rise to chronic inflammation [87].

Periodontal disease has been frequently described in patients with RA, a chronic autoimmune polyarthritis with a prevalence of 0.5% to 1.0% in the adult population and a female:male ratio of 3:1. It primarily affects joints, but is also considered to be an inflammatory systemic disease [89]. Autoantibodies such as rheumatoid factor (RF) and anti-citrullinated peptide antibodies (ACPAs) are present in 50% to 70% of RA patients, and are associated with more severe symptoms and joint damage, and increased mortality [89,90]. As in the case of other autoimmune disorders, RA seems to be due to the action of environmental factors such as smoking and microbiota on a predisposed genetic background [89]. A number of observations support an association between periodontitis and RA: RA patients have a higher prevalence of periodontitis than controls [11,12]; RA and periodontitis are chronic inflammatory conditions that share the over-expression of pro-inflammatory cytokines such as interleukin (IL)-1β, TNF-α, IL-6 and IL-8 [88]; *Porphyromonas gingivalis* (a component of the oral microbiota that is frequently associated with periodontitis) may promote aberrant citrullination (i.e., conversion of arginine to citrulline) via peptidyl arginine deiminase type IV, and eventually elicit the appearance of ACPAs [88]; the non-surgical treatment of periodontal disease is accompanied by a reduction in the severity of RA [91,92]; and periodontitis seems to negatively affect the response to RA treatment with biological agents such as TNF blockers [93].

Periodontitis also affects up to 70% of SLE patients [18]. Periodontal probing depth, a well-known marker of periodontal disease, correlates with the duration of SLE, the accumulated dose of prednisone, and serum C-reactive protein (CRP) levels [18]. A recent meta-analysis of eight case-control studies involving 487 patients with SLE and a total of 1383 participants has shown that the risk of periodontitis in the cases was markedly greater than in the controls [19].

A widened periodontal ligament space (one of the typical radiographic signs of periodontal disease) is found in about 40% of SSc patients [22,94]. It can be caused by a reduced number of periodontal capillaries together with reduced levels of vascular endothelial growth factor (VEGF) [95], as well as by increased collagen deposition [96], and may explain the high prevalence of tooth loss in SSc patients [97].

The published reports concerning the incidence of periodontal disease in patents with primary Sjögren’s syndrome are conflicting [98,99]: Earlier studies indicate an increased frequency of periodontal disease due to hyposcialia [100], but a recent study has found that the periodontal status of patients is similar to that of healthy subjects [101].

### 2.7. Jaw Claudication

Jaw claudication is defined as masticatory muscle pain on chewing due to ischemia-induced functional impairment [26]. Intermittent claudication, which extends to all skeletal muscles, is a dynamic concept in which muscle activity in the presence of a reduced blood supply such as that caused by arterial stenosis (or, in this case, vasculitis) induces muscle ischemia, an accumulation of lactate, and consequent pain.

Jaw claudication is one of the typical manifestations of the onset of giant cell arteritis, which affects the large vessels and is the most prevalent vasculitis in Western patients aged >50 years [102]. Giant cell arteritis almost exclusively occurs in Caucasians and more frequently in females (female:male ratio 3:1), and typically involves the extra-cranial branches of the carotid artery such as the temporal artery, which may appear tortuous and be tender upon palpation. A Doppler ultrasound examination usually reveals an inflammatory process. About 40% to 60% of patients with giant cell arteritis have polymyalgia rheumatica, an inflammatory disease characterized by pain and stiffness of the neck, shoulders and pelvic girdle [103]. The symptoms of giant cell arteritis include severe temporal headaches, low-grade fever, malaise, depression and weight loss. Patients may also experience dental pain, dysphagia, dysarthria, chronic cough and, albeit rarely, necrosis of the lips and tongue [26]. One of the most feared complications of giant cell arteritis is total or partial visual loss which, unless the involvement of the posterior ciliary arteries is promptly recognized, may occur in up to 20% of patients. Patients reporting jaw claudication should therefore be immediately evaluated by a rheumatologist in order to exclude giant cell arteritis and prevent serious complications [104]. Treatment with corticosteroids is usually effective, but methotrexate and tocilizumab are valid alternatives in patients who need steroid-sparing treatments [103,105].

### 2.8. Temporomandibular Joint Involvement

Inflammation of the temporomandibular joint (TMJ) is frequent in IRD patients [13]. It may restrict jaw growth in children, and lead to micrognathia, deviation to the affected side, and ankylosis; in adults, it may give rise to jaw pain, swelling, a limited range of motion, and malocclusion. Although often asymptomatic (especially in the early phase of the disease), TMJ involvement may be found in 90% of patients with juvenile idiopathic arthritis, which refers to all forms of arthritis that begin before the age of 16, persist for more than six weeks and have an unknown etiology [15,16]. On the basis of the revised 1997 International League of Associations of Rheumatology criteria, the classification of juvenile idiopathic arthritis includes seven entities (systemic arthritis, oligoarthritis, RF-positive and RF-negative polyarthritis, juvenile psoriatic arthritis, enthesitis-related arthritis, and undifferentiated juvenile idiopathic arthritis) (Table 2) [106], but the extremely heterogeneous nature of the disorders included in the definition has recently led to a call for a new classification [107]. 

Juvenile idiopathic arthritis is the most frequent childhood chronic rheumatic disease and has a prevalence of up to 150 cases per 100,000 in developed countries [108]. It is characterized by inflammatory morning stiffness lasting more than 60 min, joint swelling and tenderness, and functional impairment of the affected joints. Patients may also experience uveitis, which may be present at onset (particularly in the case of ANA-positive oligoarthritis) and, if left untreated, can lead to complications and eventual blindness [109,110]. Although TMJ arthritis normally occurs during the course of arthritis of other joints, it may also be the only presentation of juvenile idiopathic arthritis [111]. Prompt recognition is important because of the risk of severely altered cranial growth in young patients [112].

In adults with longstanding RA, TMJ symptoms develop late and discomfort is reported only when jaw motion is markedly limited [113]. TMJ involvement has been reported in up to 88% of RA patients [14], and may initially manifest itself as pain and swelling, with crepitation and stiffness upon mouth opening appearing later [114]. 

There is no conclusive laboratory test for a diagnosis of juvenile idiopathic arthritis, which is based on history, a physical examination, inflammatory markers, and the exclusion of other causes of arthritis. Only 5% of patients are positive for RF, which may be associated with ACPAs. ANAs are not specific but, in the presence of oligoarthritis, are associated with a high risk of developing chronic uveitis [110]. Conventional radiographic methods (plain X-rays or orthopantomography, depending on the stage of the disease) reveal various patterns, including erosions, flattening, reabsorption and the complete destruction of the mandibular condyle [115]. In late stages, osteophytes, bone sclerosis, and reduced joint space become evident. Computed tomography (CT) can provide more detailed images of the condyles and surrounding structures despite its limitation in differentiating active versus previous changes in joints and soft tissues. Cone beam CT has progressively replaced conventional multi-slice CT in TMJ study as it provides cheaper but accurate sub-millimeter-resolution images with less radiation exposure [116]. Magnetic resonance imaging (MRI) allows a better assessment of articular and periarticular soft tissues, and both qualitative and quantitative evaluations of synovial fluid effusion, bone marrow edema, erosions, cartilage damage, and articular disc and ligamentous involvement. The use of gadolinium contrast improves the visualization of synovial tissue (which appears enhanced due to its vascularization), erosions and cartilage changes [117]. Ultrasonography may help to identify active synovitis and guide intra-articular injections [118]. The differential diagnosis of TMJ arthritis includes osteoarthritis, villonodular synovitis, osteochondroma, trauma and infections [119].

The pharmacological treatment of juvenile idiopathic arthritis, including non-steroidal anti-inflammatory drugs (NSAIDs), corticosteroids, and conventional synthetic and biological DMARDs, improves TMJ symptoms, but additional physical medicine, jaw appliances and postural training may be useful [120]. Although intra-articular treatment with corticosteroids has long been suggested as a treatment option, its use in pediatric patients is controversial because there are concerns that its short-term benefit may be off-set by long-term adverse effects on mandibular growth [121]. If medical management is not enough to control symptoms, surgical treatment may be necessary as it is for other joints in the body [108]. 

## 3. Conclusions

Oral manifestations are very frequent in patients with rheumatic diseases and may be the reason for first consulting a doctor. Some manifestations such as oral ulcers or xerostomia are aspecific and very frequent, whereas others are pathognomonic of rare diseases, such as strawberry-like gingivitis in patients with granulomatosis with polyangiitis or microstomia in SSc patients. Rheumatic diseases may have common pathogenic mechanisms as they are the consequence of interactions between genetic factors, altered immunological responses to environmental factors, lifestyles and previously or currently received healthcare measures. Increasing knowledge of these mechanisms and interdisciplinary collaboration have improved the diagnosis and treatment of the severe oral and systemic complications of rheumatic diseases.

## Figures and Tables

**Table 1:** The main immune-mediated and inflammatory rheumatic diseases associated with significant oral manifestations.

Disease	Definition	Hallmark Biomarkers	Main Oral Manifestation	Ref.
Rheumatoid arthritis	A chronic inflammatory disease marked by symmetrical, peripheral polyarthritis	RF, ACPA	Periodontitis, TMJ involvement	[11,12] [13,14]
Juvenile idiopathic arthritis	A clinically heterogeneous group of arthritides that appear before the age of 16	ANA, RF, HLA-B27	TMJ involvement	[13,15,16]
Systemic lupus erythematosus	A chronic autoimmune disease potentially targeting any organ	ANA, anti-dsDNA, anti-Sm	Oral aphthous ulcers, periodontitis	[2,3,17] [18,19]
Sjögren’s syndrome	A chronic autoimmune inflammatory disease primarily targeting exocrine glands	ANA, anti-SSA/Ro, anti-SSB/La	Xerostomia	[20]
Systemic sclerosis	A connective tissue disease characterized by multi-system involvement (skin, lungs, cardiovascular and gastro-intestinal systems)	Anti-Scl70-topoisomerase I, anti-CENPA/B	Dysphagia, microstomia, periodontitis	[10,21,22]
Immune-mediated myopathies	A group of acquired heterogeneous autoimmune disorders characterized by muscle weakness	ANA, myositis-specific antibodies	Dysphagia	[23]
Behçet’s disease	A multi-systemic disorder characterized by oral aphthous ulcers, genital ulcers and ocular involvement	HLA-B51	Oral aphthous ulcers	[24,25]
Giant cell arteritis	Vasculitis of medium-sized and large vessels typically involving branches of the carotid artery such as the temporal artery	None	Jaw claudication	[26]
Granulomatosis with polyangiitis	Granulomatous vasculitis involving small arterial and venous vessels	cANCA (anti-PR3)	Strawberry-like gingivitis	[27,28]

RF: Rheumatoid factor; ACPA: Anti-citrullinated protein antibodies; ANA: Anti-nuclear antibodies; cANCA: Cytoplasmic anti-neutrophil cytoplasm antibodies; anti-Sm: Anti-Smith antibodies; CENP: Centromere nuclear protein A and B; HLA: Human leukocyte antigen; PR3: Proteinase 3; SSA and B: Sjögren’s syndrome antigen A and B; TMJ: Temporo-mandibular joint.

**Table 2:** International League of Associations of Rheumatology (ILAR) classification criteria for juvenile idiopathic arthritis (age at onset <16 years, duration of clinical manifestations ≥6 weeks) [107,109].

Classification	Frequency	Onset	F:M
Oligoarticular Extended Persistent	(27% to 56%)	Early childhood, peaks at 2–4 years	F > M
Polyarticular RF negative RF positive	(11% to 28%) (2% to 7%)	Late childhood or adolescence Peaks at 2–4 and 6–12 years	F > M
Systemic arthritis	(4% to 17%)	Throughout childhood	F = M
Enthesitis-related arthritis	(3% to 11%)	Late childhood or adolescence	M > F
Psoriatic arthritis	(2% to 11%)	Peaks at 2–4 and 9–11 years	F > M
Undifferentiated arthritis	(11% to 21%)	-	-

F: Female; M: Male; RF: Rheumatoid factor.
